# Supplementary material for: The critical dynamics of hippocampal seizures
Source: Nat Commun. 2024 Aug 13;15:6945. doi: 10.1038/s41467-024-50504-9 (PMC11322644; doi:10.1038/s41467-024-50504-9)
Supplement: Supplementary file 1 — Supplementary Information [file 41467_2024_50504_MOESM1_ESM.pdf]

Supplementary Information

Lepeu et al., ‘The critical dynamics of hippocampal seizures’

Supplementary Discussion

Much of the language in epileptology is intrinsically inspired from the observed dynamics of the epileptic brain. Terms such as 'seizure thresholds' or 'excitability' are broadly used, but are often vaguely defined. In the Epileptor, these terms have a mathematical meaning explained here.

Dynamical model

The Epileptor consists of five differential equations (see methods), which capture the ensemble of dynamics of the system. The model is characterized by a state-space, which is the ensemble of possible trajectories governed by the constitutive variables. The Epileptor's dynamics can be most intuitively represented by a bifurcation diagram, in this case showing a fold bifurcation (Supplementary Fig. 1).

In a mechanical analogy (stability landscapes in Fig. 1C), the system (the brain, here shown as a ball) can remain within the 'non-ictal' (1) or 'ictal' (2) basin of attraction at any time or travel the distance (4) to reach and cross the threshold (3), resulting in a critical (here ictal) transition (6, red vertical arrow). In physiological conditions (dark grey ball), the non-ictal basin is deep, preventing spontaneous ictal transitions. In the presence of small perturbations (7), the system's trajectory rapidly recovers within the non-ictal regime given the strong recall flow (8). Following the mechanical analogy, the recall flow corresponds to the slopes of the landscapes (Fig. 1C) and determines the recovery rate to perturbations. Bi-directional changes in excitability (9) reconfigure the stability landscape with lower (yellow ball) or heightened resilience and recovery rate (green ball). When the excitability reaches the critical point (empty circle), the non-ictal regime disappears and the system is forced to transition (5) to the ictal regime, resulting in an unprovoked seizure (red landscape in Fig. 1C).

A crucial insight from the study of fold bifurcations is that a latent decrease in the system's resilience typically has a minimal impact on its observable state until the point of failure. In the case of epilepsy, ictal transitions could spontaneously occur close to the critical point by threshold crossing due to stochastic fluctuations of neural activity. The system's resilience may nevertheless be assessed by actively probing it. The observation of recovery dynamics upon imposed fixed stimuli, may allow to infer varying resilience.

In our experiments, recovery is quantified and ictal transitions are forced at many levels of excitability. This allowed us to draw the contours of the stability landscapes in hippocampal circuits.

Dynamical metrics

We here provide supplementary explanation on the metrics used in the study and their significance in dynamical terms. Central to our study, we imposed perturbations to the hippocampal circuits to induce transient iEEG response and self-sustained seizures. We used weak (single-pulse) and sustained (pulse train) stimulations to induce short and long excursions within and across basins of attraction, respectively. Crucially, the length of these excursions could be measured in the recorded time series to infer recovery and resilience, respectively.

Recovery and line-length

We quantified responses to *subthreshold* stimulations as the line-length, an integrative measure of change over time (see methods). We asked whether this metric directly corresponded to a distance in the Epileptor's state space. In the model, line-length of the simulated timeseries ($x_1 + x_2$) directly reflects the path traveled in the state space, making it a reliable metric to quantify responses to and recovery from subthreshold perturbations (Supplementary Fig. 2A-B). In mice and humans, we calculated the LL over a 250ms window capturing the evoked iEEG potential upon stimulation (Supplementary Fig. H-I). As in the model, line-length changes as a function of the magnitude of the input stimulus and the underlying neural excitability (Fig. 1 and 3). Given what we have learned from the model, we interpret this measurement as an excursion length within the non-ictal basin of attraction. Importantly, it reflects the recovery rate (slope of the basin or flow) where a fast recovery leads to a shorter excursion and vice versa (inverse relationship).

Resilience and time-to-seizure

We quantified responses to *suprathreshold* stimulations as the time-to-seizure, a straightforward measure of the magnitude of perturbation needed to induce an ictal transition (see methods). We asked whether this metric reflected the distance to the seizure threshold in the Epileptor. We first showed in the model that a given time-to-seizure tightly corresponded to the cumulative line-length in the simulated timeseries (x_1+ x_2) up to seizure onset (Supplementary Fig. 2C). We then showed that this cumulative line-length corresponded to the path traveled in the state-space of the model (Supplementary Fig. 2E). More specifically, the cumulative line-length directly reflects the excursion within the non-ictal basin up to the seizure threshold (here defined as neural resilience, the 'height of the hill', Supplementary Fig. 2D-E). Of note, the excursion length does not only depend on the underlying degree of excitability, but also on the stimulation frequency (Supplementary Fig. 2F). Indeed, when stimulation frequency is low, the system has more time to recover between incoming perturbations, and therefore the path is longer (due to back and forths forward trajectories). Thus, in the model, the cumulative line-length and the time-to-seizure directly reflect neural resilience for a given stimulation frequency. When the excitability reaches the critical point, both metrics tend to zero: the system will transition to seizure even in absence of external perturbations.

In mice, we used optogenetic stimulations, which has the advantage of enabling the measurement of iEEG responses during a stimulation train, as opposed to simulations using electrical current (artifacts). We could show that the cumulative line-length to seizure varies as a function of neural excitability in mice with a mean difference with NaCl condition [bootstrapped 95%CI] of +71% [+53,+97] in presence of BZD and -19% [-5,-30] in presence of PTZ (Supplementary Fig. 2J-K). For given stimulation parameters (here 20Hz), time-to-seizure is highly correlated with cumulative line-length ($r2=0.85$, $p<0.005$, Supplementary Fig. 2L) justifying its use to practically measure resilience to seizure in mice and humans in our main analysis (Fig. 2).

Observed seizure dynamics in mice

To study and compare the onset and offset dynamics of the two seizure models in mice, we applied a spectral and a visual analysis to classify dynamotypes[1]. Among seven mice that had 15 seizures provoked by optogenetics (Supplementary Fig. 4A) and 14 seizures after receiving a PTZ injection (Supplementary Fig. 4B), we found that the spectral profile of these seizure onsets and offsets was not different (Supplementary Fig. 4C-E).

To gain more precise insight into the exact pairs of bifurcations crossed at the onset and offset of provoked and unprovoked seizures, we visually reviewed each traces according to the methodology proposed by Saggio et al[1]. Briefly, this review was based on the visual identification of scaling laws or the absence thereof in the ictal spikes amplitude or frequency at the onset and offset of each seizure. This visual review could not be blinded, as provoking stimulations are visible in EEG traces.

We found that the onset bifurcation was either a saddle-node (SN) or a saddle-node on invariant cycle (SNIC, Supplementary Table 2). SN and SNIC bifurcations are both fold bifurcations captured by the Epileptor, with one notable difference: SNIC have a brief acceleration (reducing inter-spike intervals) whereas SN have a fixed frequency at the onset of the seizure. There was no significant difference in the proportion of the SN onset between provoked and unprovoked seizures (6/15 vs. 10/14, $p=0.18$, Chi-square test, Supplementary Table 2). In the healthy hippocampus of our mice, we never observed a supercritical Hopf bifurcation at seizure onset, also commonly named low-amplitude fast activity (LAFA). Of note, saddle-node cannot theoretically always be distinguished from a subcritical Hopf bifurcation (SubH), and our lack of DC-recordings made this distinction impossible in our observational data. We next experimented by varying parameters of the provoking optogenetic stimulation to rule the possibility of a subcritical Hopf onset bifurcation (see below).

We found that the offset bifurcation was either a saddle-homoclinic (SH), a saddle-node on invariant cycle (SNIC), a supercritical Hopf (SupH) or a subcritical Hopf (SubH). The Epileptor model captures a SH offset bifurcation, which is difficult to distinguish from a SNIC offset bifurcation, as both show

slowing-down of a few ictal spikes, the former with a logarithmic and the latter with a root-square scaling law. The Epileptor does not account for SupH or SubH offset bifurcations observed with equal proportion in provoked and unprovoked seizures (8/15 vs. 7/14 , p=0.41, Chi-square test, Supplementary Table 2).

Probed seizure onset dynamics in mice

Our focus was on seizure onset. To verify that the observed dynamics were a SN or a SNIC and not a SubH, we varied the provoking stimulation in a number of ways. SN and SNIC are bifurcations of the integrator type, that is weak perturbations may accumulate to push the system over the bifurcation point (Supplementary Fig. 5A). On the other hand, SubH are of the resonator type, in which weak perturbations at the 'resonance frequency' may provoke a bifurcation crossing, but weak perturbations at other frequencies will not.

As with Epileptor simulations (Supplementary Fig. 5B), we found in our experiments that a broad range of optogenetic stimulation frequencies (4-40Hz) could provoke seizures in the healthy hippocampus of our mice. None of these frequencies seemed to resonate with the system, but rather perturbations accumulated at a different rate until meeting a threshold (Supplementary Fig. 5C). Indeed, optogenetic stimulations delivered non-rhythmically but at a given rate per second were as efficient as rhythmic stimulations to provoke seizures (Supplementary Fig. 5B4, C4, F). Also, optogenetic stimulation of inhibitory PV-interneurons (see methods) from 4 to 100 Hz did not provoke seizures, although it entrained the hippocampal oscillation at these frequencies.

From the above observations and these experiments, we concluded that the healthy hippocampus harbors a seizure onset bifurcation with integrator properties, as captured by the Epileptor model.

Supplementary Figures

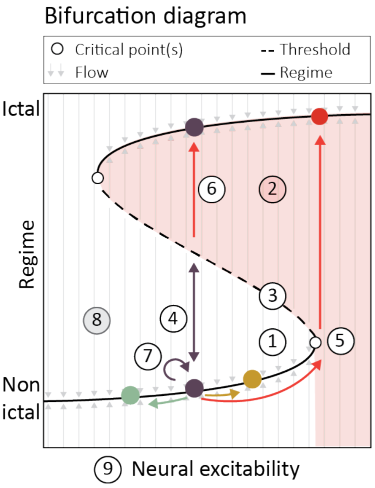

Dynamical system	Epilepsy
1 Basin of attraction 1	Non-ictal regime
2 Basin of attraction 2	Ictal (seizure) regime
3 Threshold or separatrix	Seizure threshold
4 Distance to threshold	Neural resilience
5 Critical point crossing	Unprovoked seizure
6 Threshold crossing	Provoked seizure
7 Perturbation response	Evoked response (potential)
8 Flow or gradients	Recovery rate
9 Control parameter	Neural excitability

Supplementary Figure 1: Epileptor model and terminology. Left: Bifurcation diagram of neural excitability in the Epileptor showing a fold bifurcation that summarizes important concepts of nonlinear dynamics. **Right**: corresponding terminology in dynamical systems theory (left column) and epileptology (right column).

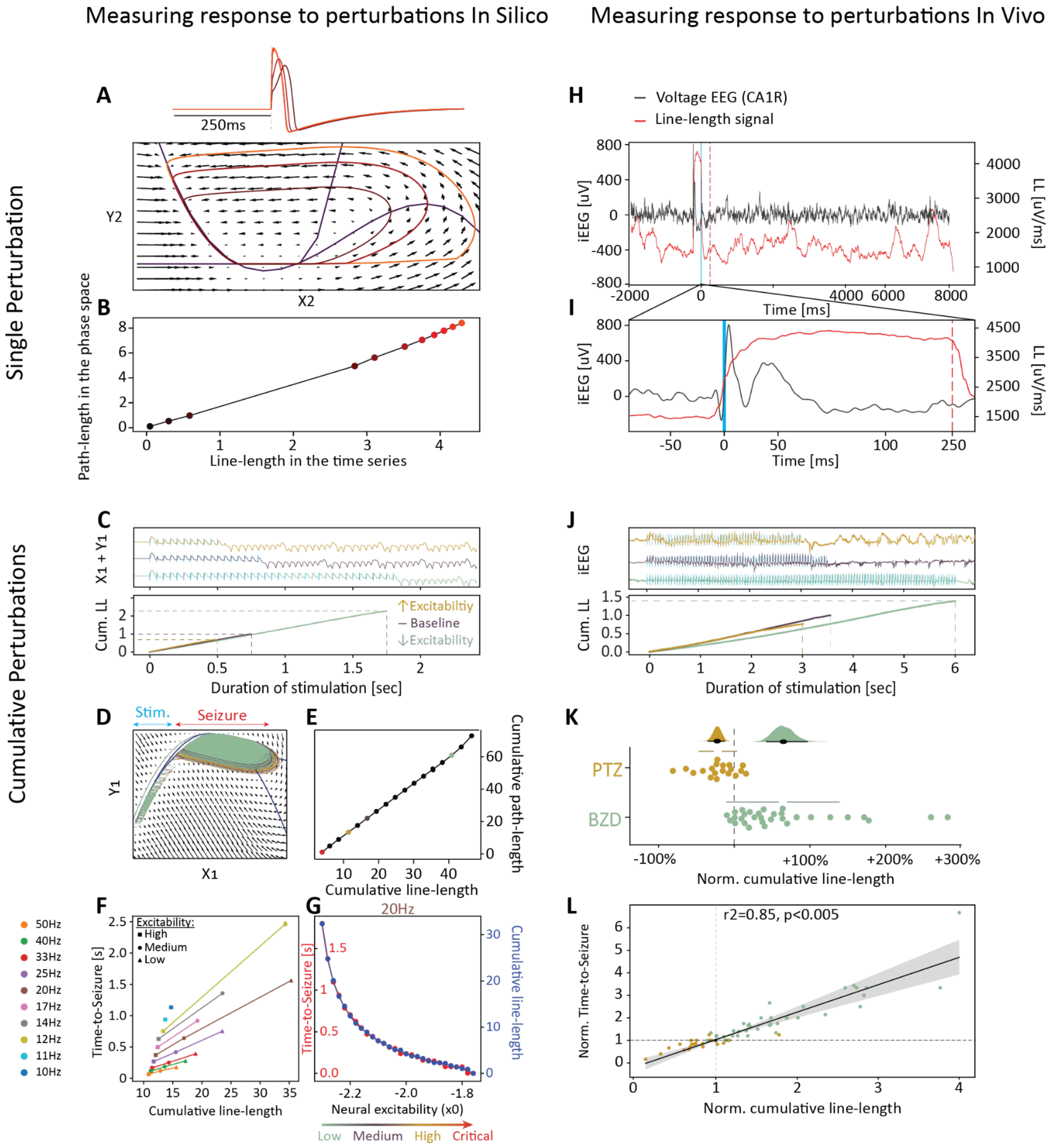

Supplementary Figure 2: Dynamical model and metrics. A: In the Epileptor, small perturbations (single-pulse applied on subsystems 1 and 2) lead to variable responses in subsystem 2. Three color-coded trajectories are shown in subsystem 2 (y_2 versus x_2, spike-wave discharge, bottom) and correspond in the timeseries to evoked responses to a single-pulse stimulation at different intensities (x_1+ x_2, top). Nullclines (derivative = 0) are shown in violet, and vectors show the instantaneous gradients (derivative ≠ 0). **B:** Correspondence between the line-length measured in the time-series and the path-length in the state-space. Colored dots (as in A and main Fig. 1E1) correspond to stimulation at different intensities. **C:** Sustained stimulations lead to cumulative perturbations which can reach the seizure threshold. Upper panel shows timeseries of 20Hz train stimulation (pulses as cyan ticks) leading to the onset of a seizure in Epileptor. Lower panel shows the cumulative line length over time (Cum. LL) derived from these traces up to the seizure onset. Bi-directional changes in the neural excitability (color-coded, same as in Fig. 1-3) lead to changes in the amount of perturbations (cumulative line length) needed to reach the threshold. **D:** Trajectories of provoked seizures in the

state space of the Epileptor's subsystem 1 (y_1 versus x_1, high-frequency activity). Each stimulation (small loop on the left) moves the system toward the threshold, and when reached results in a self-sustained seizure (larger loops on the right). Nullclines and vectors as in A. **E:** Correspondence between the cumulative line-length measured in the time-series and the path-length in the state-space as in B, but for train stimulations. **F:** Correspondence between the cumulative line-length and time-to-seizure for different excitability levels (marker shape) and stimulation frequency (color-coded). **G.** Correspondence between cumulative line-length and time-to-seizure tested at 20 Hz as a function of increasing excitability levels. **H:** Line-length (LL) measured *in vivo* from the evoked iEEG potential, (here right CA1 hippocampus) in response to single-pulse stimulation (cyan line) in the entorhinal cortex of one mouse. **I:** LL calculated on a 250ms window (red dash line). **J:** As in C, but for three seizures induced in one mouse at different excitability levels (GABAergic drugs, color-coded). **K:** Cumulative line-length to seizure as a function of neuronal excitability in mice. Mean difference with NaCl condition [bootstrapped 95%CI]. **L:** Correspondence between time-to-seizure and cumulative line-length across mice and pharmacological conditions, normalized to the control condition (NaCl).

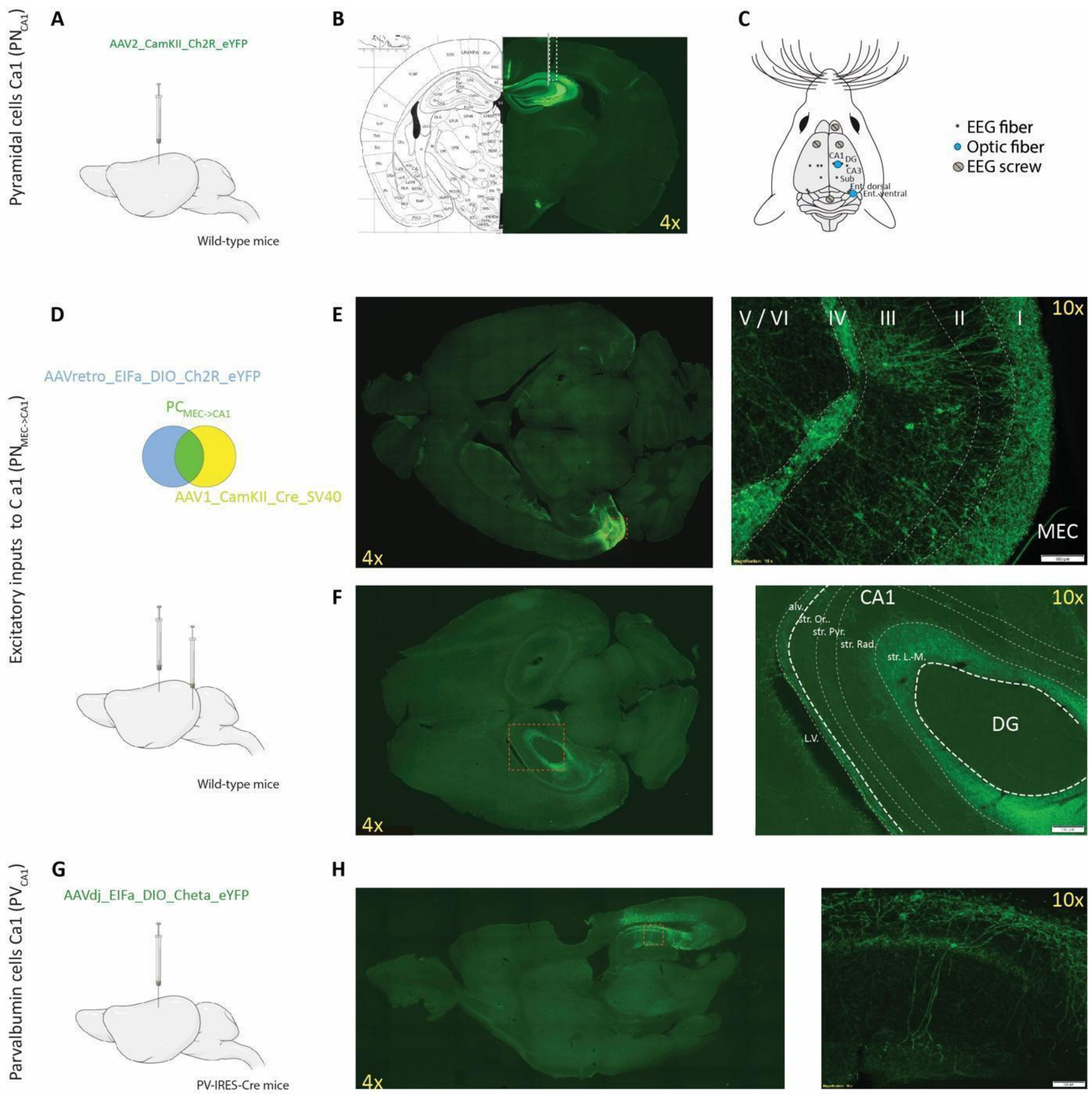

Supplementary Figure 3: Viral transfections and electrodes placement

A-B: Expression of Channelrhodopsin (Ch2R) under CamK2 promotor in the pyramidal neurons of the right dorsal CA1 hippocampus (B, $PN_{CA1,}$ coronal view) after injection of 450nl of AAV2_CamK2_Ch2R_eYFP (A). White dashed lines and the gray line indicate the location of the optic fiber and the CA1 electrode glue to it, respectively. **C:** Three weeks after viral injection, mice were implanted with 12 multisite iEEG electrodes as well as an optic fiber on the stimulation site, either the CA1 hippocampal cortex for PN_{CA1} and PV_{CA1} or the entorhinal cortex for $PN_{MEC\text{->}CA1}$. **D-F:** Conditional expression of Ch2R under CamK2 promotor (D) in the layer III pyramidal neurons of the right medial entorhinal cortex (E, axial view) projecting to the stratum lacunosum moleculare of the ipsilateral dorsal CA1 hippocampus (F, $PN_{MEC\text{->}CA1}$, axial view). The intersectional viral strategy (D) ensured that only neurons projecting from the entorhinal cortex to the ipsilateral CA1 expressed the ChR2. **G-H:** Expression of a fast Channelrhodopsin (Cheta) in parvalbumin-positive interneurons of the dorsal CA1 hippocampus of PV-IRES-Cre mice (H, sagittal view). Right panels show 10x zoom corresponding of the red square in the left panels. Panels A, D and G created with BioRender.com released under a Creative Commons Attribution-NonCommercial-NoDerivs 4.0 International license https://creativecommons.org/licenses/by-nc-nd/4.0/deed.en.

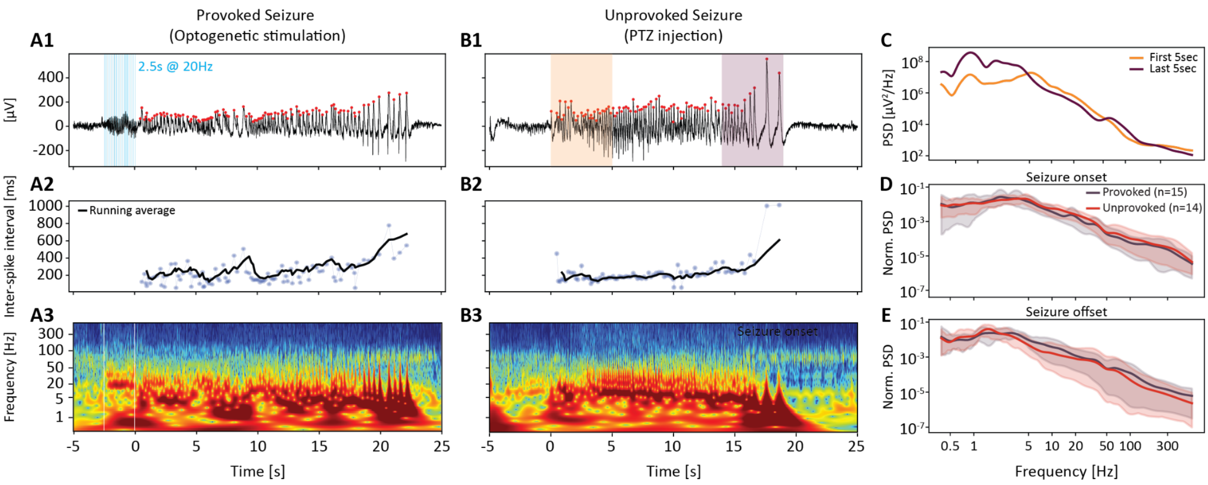

Supplementay Figure 4: Comparison between provoked and unprovoked seizure in mice. A1-A3: Example of optogenetically provoked seizure (2.5s of 20Hz stimulation of $PN_{MEC->CA1}$), recorded in CA1. **B1-B3:** In the same mouse and same electrode, example of an unprovoked seizure following a suprathreshold PTZ injection (35mg/kg). Both seizures started with epileptic spike (red dots in top panel) around 5Hz with high frequency activity nested in-between them. No scaling low was observed in the inter-spike interval (middle panel) at the seizure onset. Seizures lasted around 20 seconds, and finished by a slowing down in the spike frequency. **C.** Periodogram of both the seizure onset (first 5s) and offset (last 5s), corresponding to the shaded areas in B1. **D.** Comparison of the seizure onset (first 5s) among a subset of 7 animals which underwent both optogenetic seizure induction and subconvulsive PTZ injection (done on different days, in a randomized order). Both types of seizure show similar spectral profiles. **E.** Same comparison for the seizure offset (last 5s).

A

	Integrators	Resonators
**Characteristics**	-> No frequency preference<br>-> No Inhibition-induced transition	-> Frequency preference<br>-> Inhibition-induced transition
**Type of Bifurcations**	-> Saddle-Node<br>-> Saddle-Node on invariant cycle	-> Supercritical Andronov-Hopf<br>-> Subcritical Andronov-Hopf

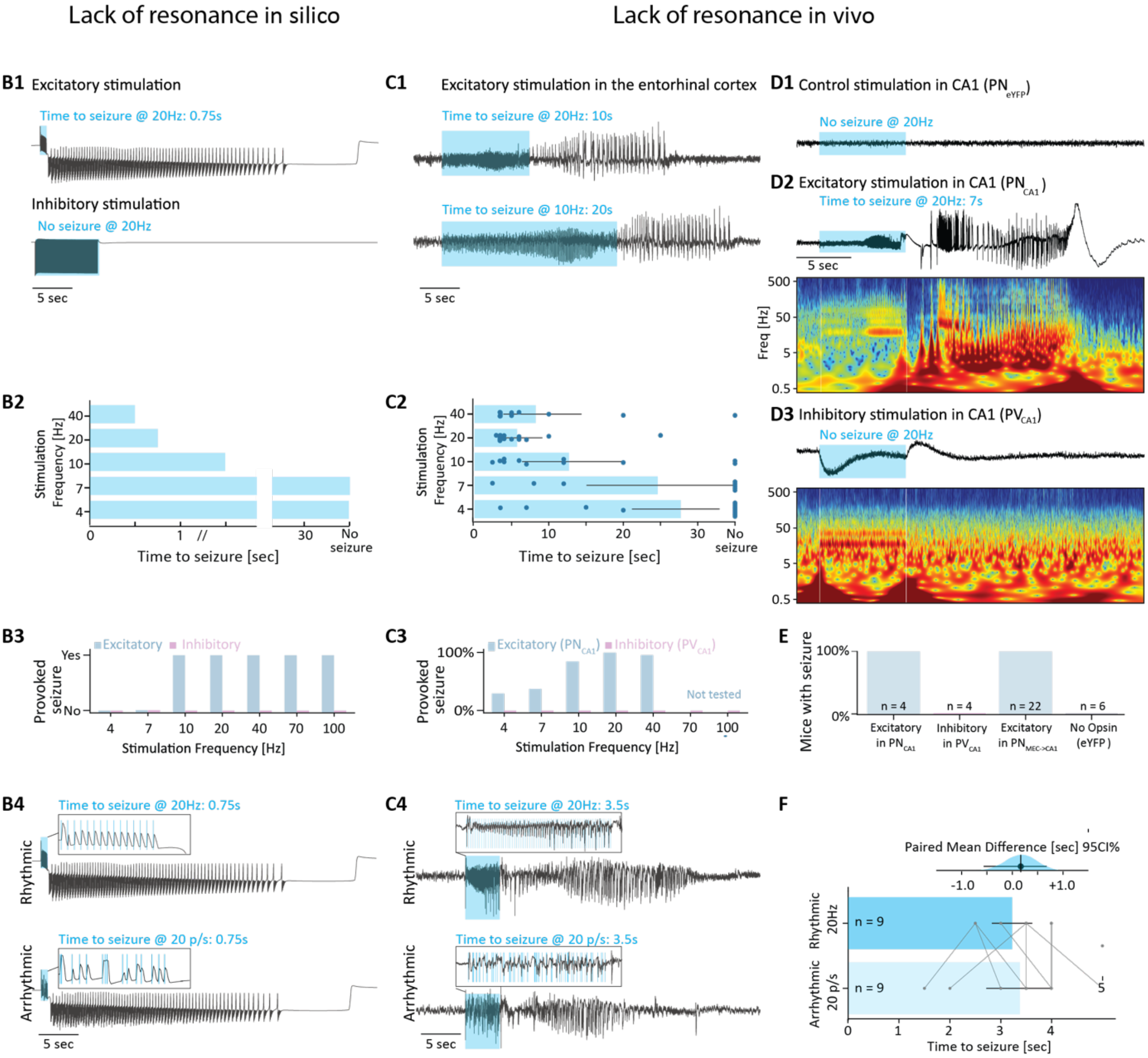

Supplementray Figure 5: Integrative dynamics in the hippocampus. A: Table of the four possible critical transitions and their characteristics, adapted from Izhikevich[2]. **B1-3:** In the Epileptor model, seizures could be induced using trains of excitatory stimulations applied to subsystems 1 and 2, but not with inhibitory stimulations (4-100Hz, train duration between 0.25-30s). **B4:** Arrhythmic stimulations (same number of pulses per second but with random inter-pulse intervals) could also elicit seizure and with similar time-to-seizure. **C-F**: Verification of the Epileptor prediction in awake non-epileptic mice. In pyramid neurons of the entorhinal cortex, seizure could systematically be induced at 10, 20 and 40Hz in all tested mice (**C2**, N=8, higher frequencies not tested). The time to seizure was inversely proportional to the rate of stimulation, as the integrator threshold was reached more or less rapidly (**B2** and **C2**). For frequency below 10Hz, seizure could not be elicited in every mouse (**C3**, tested with simulation up to 30s). Hippocampal seizures could be induced by both direct optogenetic excitation of the CA1 pyramidal cells (**D2**, PN_{CA1}) or by upstream stimulation of entorhinal excitatory inputs (**C1**, $PN_{MEC\text{->}CA1}$) but not by inhibition (**D3**, PV_{CA1}: local parvalbumin

interneurons). In the absence of the opsin (eYFP), no iEEG activity nor seizure could be provoked (D1). As PV-interneurons are typically fast spiking cells, we tested also high-frequency stimulation (up to 100Hz) using a faster variant of the Channelrodospin (ChETA) but couldn't induce any seizure (**C3** and **D2**). Irregular stimulations (20 pulse per seconds) also systematically induced seizure, with a time to seizure comparable to rhythmic 20Hz stimulation (example in **C4**, quantification in **F**).

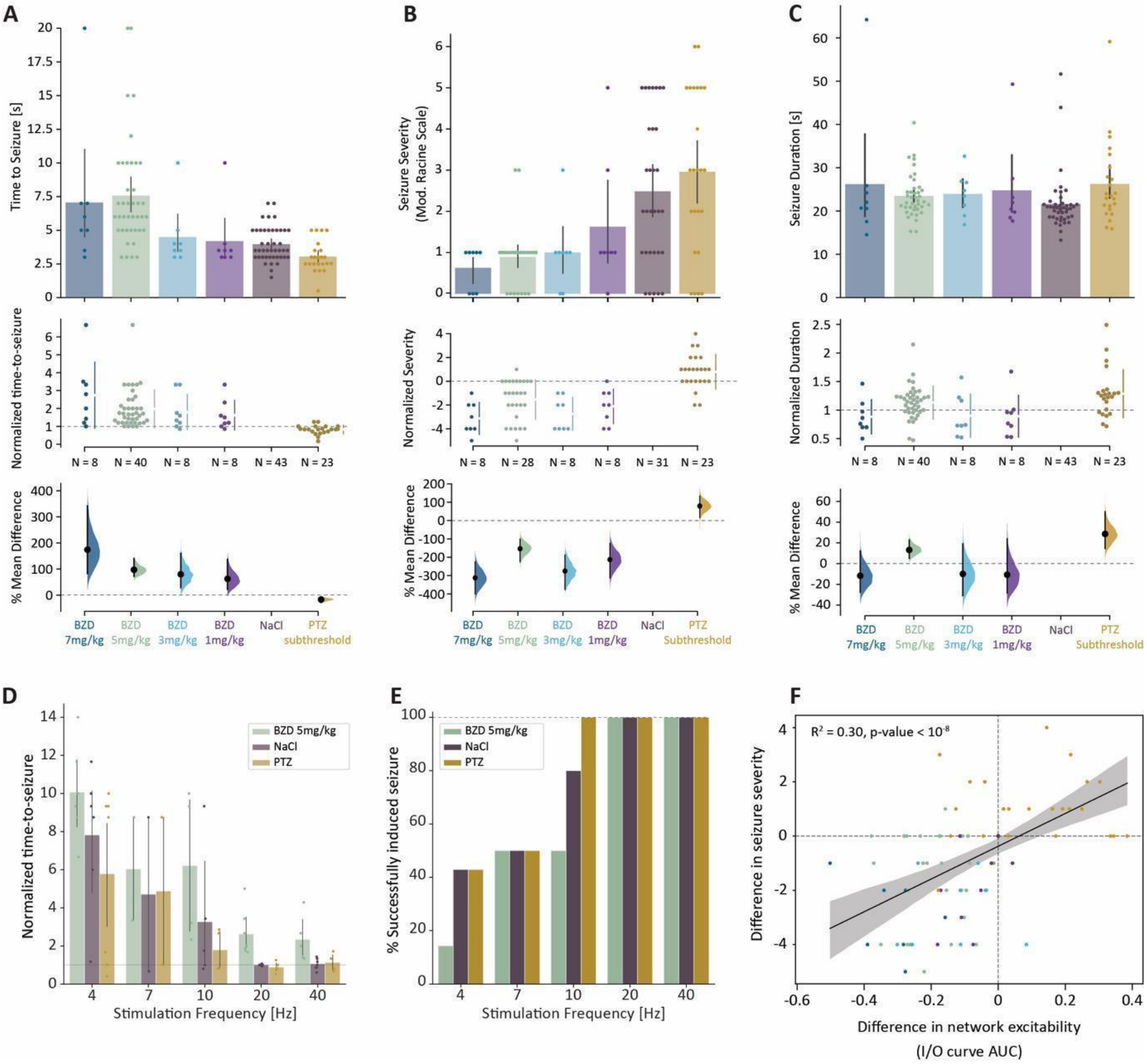

Supplemtary Figure 6: Time-to-seizure and seizure severity in mice. A-C: Top panel shows absolute values, the middle panel normalized values to control condition (NaCl), bottom panel mean difference with bootstrapped 95%CI (vertical black bar). Severity is scored using a modified Racine scale (see methods), duration is calculated from the end of the stimulation until the end of the seizure. Mean difference with NaCl in time-to-seizure: BZD 7mg/kg +174% [+82,+336], BZD 5mg/kg +97% [+72,+140], BZD 3mg/kg +80% [+29,+159], BZD 1mg/kg +62% [+21,+136] and PTZ -17% [-8,-28]. Mean difference in seizure severity: BZD 7mg/kg: -312% [-400,-225], BZD 5mg/kg -150% [-225,-96], BZD 3mg/kg -275% [-375,-188], BZD 1mg/kg -213% [-313,-97], PTZ +96% [+42,+146]. **D:** Pharmacological effect on Time-to-seizure across stimulation frequencies. The BZD and PTZ effect on resilience seems to be independent of the frequency. Only mice which were stimulated at all frequencies were included here (n=7). **E:** Percentage of sessions where seizures were successfully induced by train stimulation (max 30s). For frequencies ≥ 20Hz, seizures could always be induced, independently of the pharmacological condition. For lower frequencies, the level of excitability could have an impact on the possibility to induce seizure (i.e. the ability to reach the seizure threshold). **F:** Seizure severity directly

correlates with network response to single-pulse stimulation done minutes prior to the seizure induction.

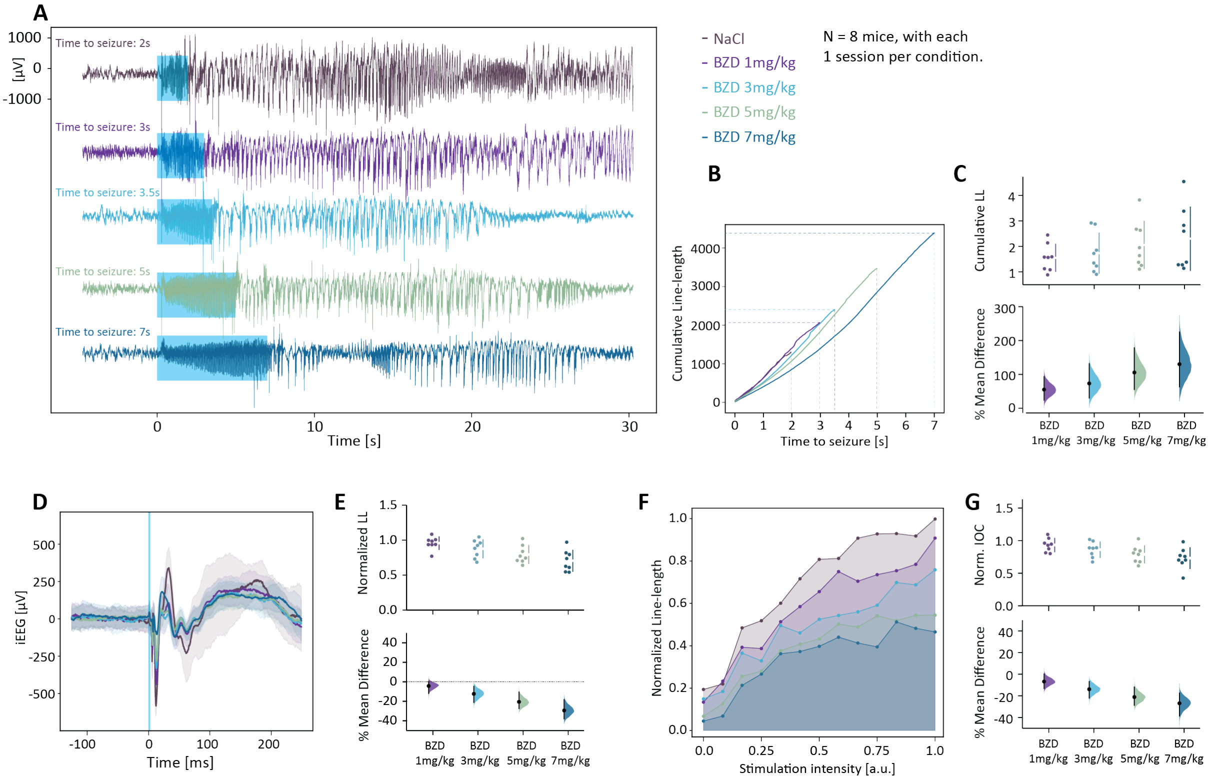

Supplementary Figure 7: Neural excitability and resilience under benzodiazepine. A. An example, in one representative mouse, of seizures induced by a 20Hz train stimulation for different doses of benzodiazepine (diazepam, BZD). **B.** Neural resilience is measured as either the time-to-seizure or the cumulative line-length to seizure. **C.** Quantification of cumulative line-length across mice and corresponding mean difference compared to NaCl [bootstrapped 95%CI]: BZD 1mg/kg: +55% [+24,+93], BZD 3mg/kg: +73% [+30,+132], BZD 5mg/kg: +106%[+55,+170], BZD 7mg/kg: +130% [+63,+225)]. Quantification of time-to-seizure is in Fig. 2C3. **D.** Neural response to small perturbation (optogenetic single-pulse, max intensity) for different levels of benzodiazepine. **E.** Quantification of the response to perturbation across mice. Mean difference with NaCl condition and 95%CI: BZD 1mg/kg -7% [0,-18], BZD 3mg/kg -17% [-9,-27], BZD 5m/kg -24% [-14,-34], BZD 7mg/kg -36% [-27,-47] **F.** Input-output curve of the iEEG response for different stimulation intensity. **G.** Quantification of the area under the input-output curve (IOC) and corresponding bootstrapped estimation statistics. Mean difference with NaCl condition and 95%CI: BZD 1mg/kg -7% [0,-13], BZD 3mg/kg -14% [-7,-22], BZD 5mg/kg -21% [-12,-29], BZD 7mg/kg -27% [-17,-39].

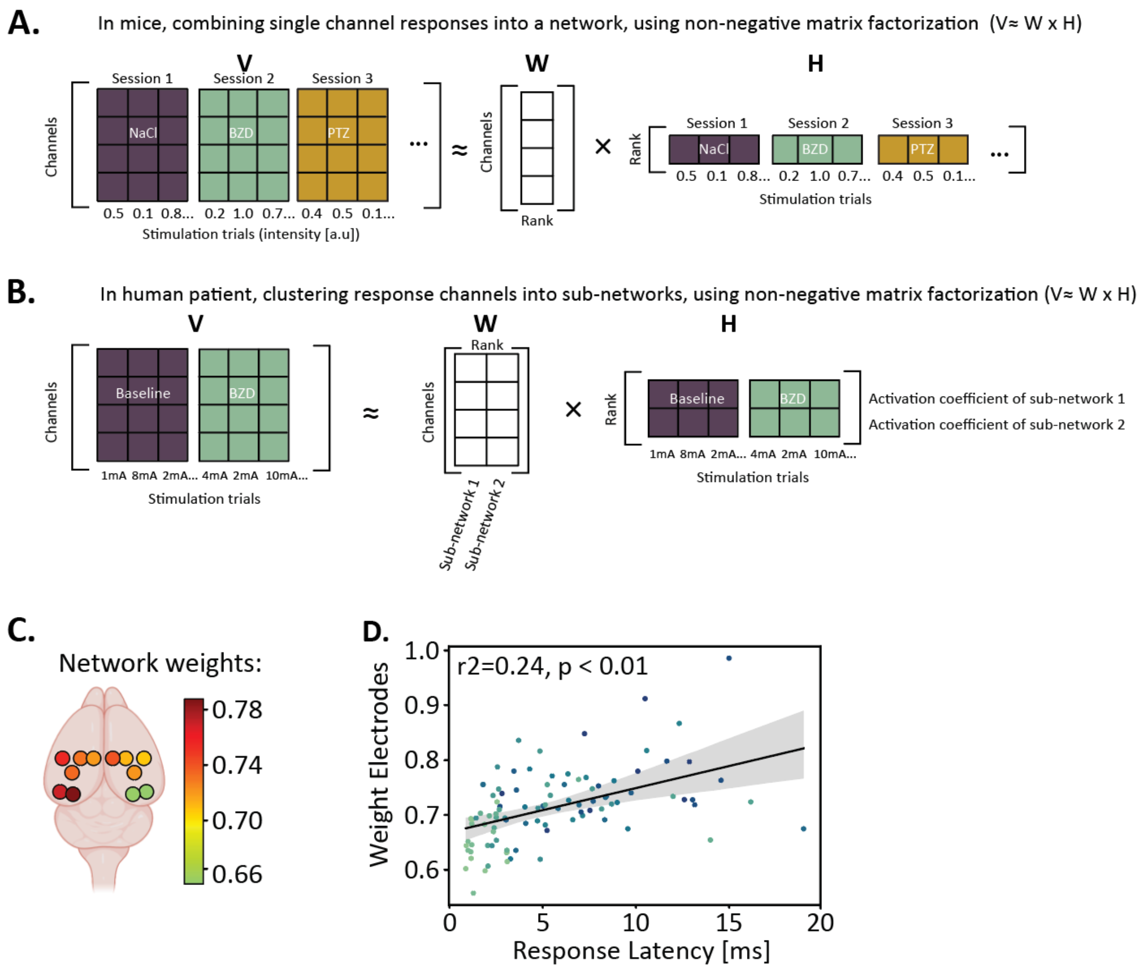

Supplementary Figure 8: Non-negative matrix factorization. A-B. Schematic of Non-negative Matrix Factorization (NMF) applied to mouse and human data, respectively. **C.** Mean weights attributed by the NMF analysis to each electrode. **D.** Positive correlation between the network weights assigned by the NMF and the latency of the response to single-pulse, suggesting a cumulative modulation by GABAergic drugs in polysynaptic connections. Each dot represents one electrode in one mouse. Panel C created with BioRender.com released under a Creative Commons Attribution-NonCommercial-NoDerivs 4.0 International license https://creativecommons.org/licenses/by-nc-nd/4.0/deed.en.

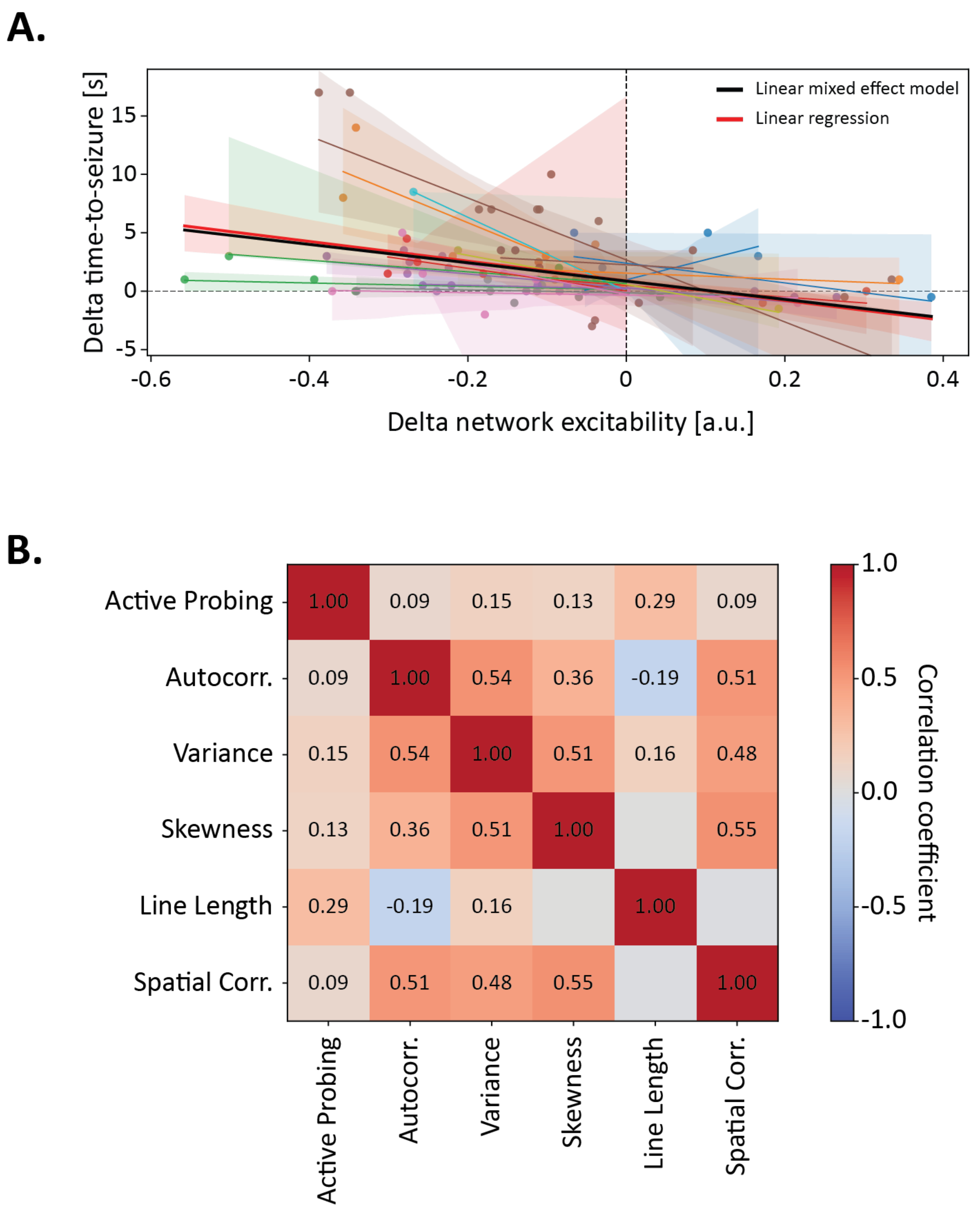

Supplementary Figure 9: Multilevel statistics & metrics correlations. A. To account for the fact that multiple observations were taken from the same animals, we used a linear mixed effect model to redo the linear regression between time-to-seizure and the network excitability (fixed effect) done in Fig. 6F, taking this time into account the different animals (random effects, allowing for both random slopes and random intercepts). The relationship between the resilience and the excitability is quite similar across animals (colors dots and thin lines) and the regression based on the linear mixed effect model (thick black line) is really similar to one obtained with a simple linear regression (thick red line, same as in Fig. 6F). **B.** For each single observation, correlation between the single pulse response and the different passive metrics on a 4-8s window after the pulse. A significant but rather weak

correlation between active and passive metrics is found, strengthening the idea that active probing adds important additional information. Interestingly, the line-length computed in absence of stimulation is the passive metric which correlated the most with active probing.

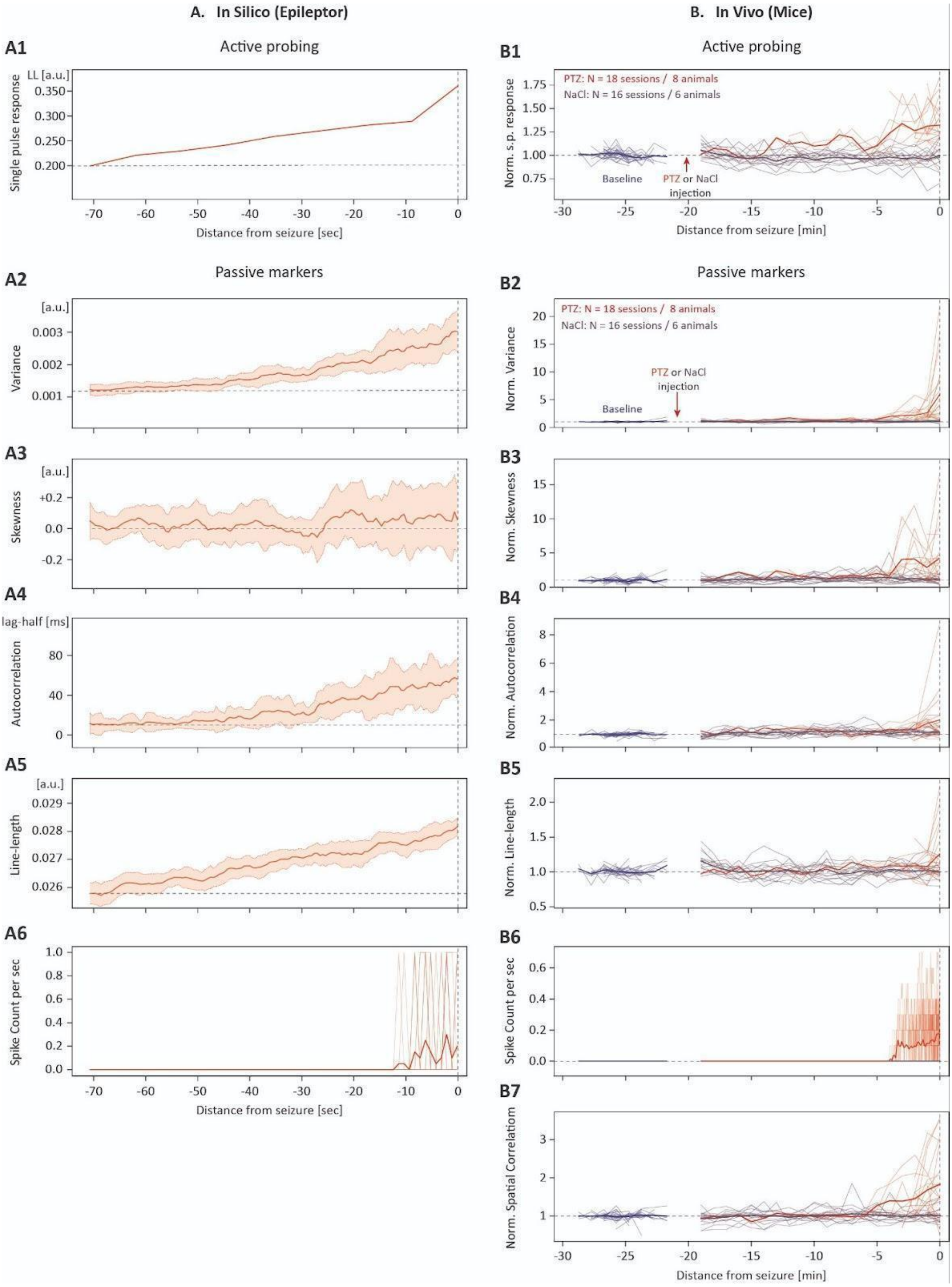

Supplementary Figure 10: Active and passive warning signs of ictal transitions. A: Warning signs of ictal transitions calculated in the Epileptor model. **A1:** As the system approaches the critical point (i.e.

the ictal transition is imminent), the response to single-pulse stimulation increases, here quantified as the line-length of the signal. **A2-A5:** When stochastic noise is added, passive dynamical signatures of critical transitions also increase when approaching the critical point. Red thick lines are the means across 20 simulations, shadings are standard deviation. **A6:** At the vicinity of the critical point, spontaneous epileptic spikes appear, revealing the instability of the system. **B:** Confirmation *in vivo* in 18 sessions among eight non-epileptic mice receiving a single convulsive dose of PTZ i.p. **B1:** Ipsilateral CA1 hippocampal responses for single-pulse optogenetic stimulation in the entorhinal cortex during the shift in excitability. After the PTZ injection, the response to single-pulse increases until the seizure occurs. Thin lines show responses for individual sessions, the thick line is the average. For each session, values are normalized to the mean response during baseline. **B2-B7:** Pre-ictal increases in passive dynamical signatures computed from the CA1 hippocampus. After supra-threshold PTZ injections, all of the passive signatures showed a significant increase just before the seizure but this was restricted to the very last moments, minutes after a visible increase in single-pulse responses (B1). **B6:** As in the model, spontaneous epileptic spikes appear just prior to the seizure.

Supplementary Table 1: Patient and seizure characteristics

N	ID	Age Gender	Etiology	Seizure onset zone	#	Single-pulses	Sz #	Stimulation Site	Semiology	Duration of stimulation [s]				
										1	2	3	4	6
1	EL003	33 F	Unknown	R Temporal	8	✓	Not tested							
2	EL004	38 M	Unknown	R subcentral gyrus	11	✓	Not tested							
3	EL005	26 F	Post-traumatic	L superior temporal gyrus	9	✓	Not tested							
4	EL008	20 F	Post-encephalitis	R Hippocampus	4	✓	1	L Hpc	Atypical	✗	✓	-	-	-
						✓	2	R Hpc	Typical	✗	✗	✓	-	-
5	EL010	45 F	Low grade tumor (DNET)	L Mesiotemporal	6	✓	1	Pre central	Atypical	✗	✗	✗	✓	-
6	EL011	19 M	Hippocampal sclerosis	L Mesiotemporal	8	✗	1	L Ent	Typical	✗	✓	-	-	-
							2	L Ent	Typical	✗	✓	-	-	-
7	EL012	56 F	Unknown	L Temporal	5	✓	1	L Hpc	Atypical	✓	-	-	-	-
8	EL014	42 M	Hippocampal sclerosis	R Temporal	6	✓	1	L Hpc	Typical	✗	✓	-	-	-
9	EL015	49 M	Hippocampal sclerosis	L Hippocampus	9	✗	1	L Ent	Typical	✗	✗	✓	-	-
							2	L Ent	Typical	✗	✗	✓	-	-
							3	L Ent	Typical	✗	✗	✓	-	-
							4	L Ent	Typical	✗	✗	✓	-	-
10	EL017	48 M	Post-hemorrhage	L Temporal pole	8	✗	1 w/o BZD	L Ent	Typical	✗	✗	✓	-	-
							2 With BZD	L Ent	Typical	✗	✗	✗	✓	-

#: Number of electrode leads, each with 8-18 contacts. Hpc: hippocampus. Ent: Entorhinal cortex, Cx: Cortex. ✗: Seizure did not start. ✓: Seizure started and was self-sustained. Typical: typical seizure semiology for subject. Atypical: unusual seizure semiology for subject. DNET: dysembryoplastic neuroepithelial tumor.

Supplementary Table 2. Seizure onset and offset dynamics in mice.

	Onset (5s)			Offset (5s)			
File	Onset Freq scaling	Onset Amp scaling	Onset bifurcation	Offest Freq scaling	Offest Amp scaling	Offset bifurcation	Condition
Ent_CamK2_59_S01	Yes	No	SNIC	No	Yes	SH or SNIC	Opto
Ent_CamK2_59_S02	No	No	SN or SubH	No	Yes	SupH	PTZ
Ent_CamK2_59_S03	Yes	No	SNIC	No	Yes	SupH	Opto
Ent_CamK2_59_S04	No	No	SN or SubH	No	Yes	SupH	PTZ
Ent_CamK2_59_S05	Yes	No	SNIC	No	Yes	SupH	Opto
Ent_CamK2_59_S06	Yes	No	SNIC	No	Yes	SupH	PTZ
Ent_CamK2_60_S03	Yes	No	SNIC	Yes	No	SH or SNIC	Opto
Ent_CamK2_60_S04	No?	No	SN or SubH	Yes	No	SH or SNIC	PTZ
Ent_CamK2_61_S01	No	No	SN or SubH	No	Yes	SupH	Opto
Ent_CamK2_61_S02	No	No	SN or SubH	Yes	No	SH or SNIC	PTZ
Ent_CamK2_61_S03	Yes	No?	SNIC	No	Yes	SupH	Opto
Ent_CamK2_61_S05	Yes	No	SNIC	No	Yes	SupH	Opto
Ent_CamK2_61_S07	Yes	No	SNIC	Yes	No	SH or SNIC	PTZ
Ent_CamK2_62_S01	No	No	SN or SubH	No	Yes	SupH	Opto
Ent_CamK2_62_S02	No	No	SN or SubH	No	Yes	SupH	PTZ
Ent_CamK2_62_S03	No	No	SN or SubH	Yes	No	SH or SNIC	Opto
Ent_CamK2_62_S04	No	No	SN or SubH	No	No	SubH	PTZ
Ent_CamK2_62_S05	Yes	No	SNIC	No	No	SubH	Opto
Ent_CamK2_62_S06	Yes	No	SNIC	No	No	SubH	PTZ
Ent_CamK2_63_S01	Yes	No	SNIC	No	Yes	SupH	Opto
Ent_CamK2_63_S02	No	No	SN or SubH	Yes	No	SH or SNIC	PTZ
Ent_CamK2_63_S03	Yes	No	SNIC	Yes	No	SH or SNIC	Opto
Ent_CamK2_63_S04	No	No	SN or SubH	Yes	No	SH or SNIC	PTZ
Ent_CamK2_63_S05	No	No	SN or SubH	No	No	SH or SNIC	Opto
Ent_CamK2_63_S06	Yes	No	SNIC	No	No	SubH	PTZ
Ent_CamK2_64_S01	No	No	SN or SubH	Yes	No	SH or SNIC	Opto
Ent_CamK2_64_S02	No	No	SN or SubH	Yes	No	SH or SNIC	PTZ
Ent_CamK2_64_S03	No	No	SN or SubH	Yes	No	SH or SNIC	Opto
Ent_CamK2_64_S04	No	No	SN or SubH	Yes	No	SH or SNIC	PTZ

Supplementary References

1. Saggio, M. L. *et al.* A taxonomy of seizure dynamotypes. *Elife* 1–56 (2020) doi:https://doi.org/10.7554/eLife.55632.
2. Izhikevich, E. M. *Dynamical Systems in Neuroscience: The Geometry of Excitability and Bursting*. (The MIT Press, 2006). doi:https://doi.org/10.7551/mitpress/2526.001.0001.
