# Supplementary material for: The critical dynamics of hippocampal seizures
Source: Nat Commun. 2024 Aug 13;15:6945. doi: 10.1038/s41467-024-50504-9 (PMC11322644; doi:10.1038/s41467-024-50504-9)
Supplement: Supplementary file 3 — Description of Additional Supplementary Files [file 41467_2024_50504_MOESM3_ESM.pdf]

File name: Supplementary Movie 1

Description: Representative example of an optogenetically induced seizure (20Hz, 3.5s) in non-epileptic mice.
