# Supplementary material for: The critical dynamics of hippocampal seizures
Source: Nat Commun. 2024 Aug 13;15:6945. doi: 10.1038/s41467-024-50504-9 (PMC11322644; doi:10.1038/s41467-024-50504-9)
Supplement: Supplementary file 5 — Reporting Summary [file 41467_2024_50504_MOESM5_ESM.pdf]

nature portfolio

Corresponding author(s): Gregory Lepeu
Maxime O. Baud

Last updated by author(s): Jun 4, 2024

Reporting Summary

Nature Portfolio wishes to improve the reproducibility of the work that we publish. This form provides structure for consistency and transparency in reporting. For further information on Nature Portfolio policies, see our Editorial Policies and the Editorial Policy Checklist.

Statistics

For all statistical analyses, confirm that the following items are present in the figure legend, table legend, main text, or Methods section.

n/a	Confirmed	
☐	☒	The exact sample size (n) for each experimental group/condition, given as a discrete number and unit of measurement
☐	☒	A statement on whether measurements were taken from distinct samples or whether the same sample was measured repeatedly
☐	☒	The statistical test(s) used AND whether they are one- or two-sided<br>*Only common tests should be described solely by name; describe more complex techniques in the Methods section.*
☐	☒	A description of all covariates tested
☐	☒	A description of any assumptions or corrections, such as tests of normality and adjustment for multiple comparisons
☐	☒	A full description of the statistical parameters including central tendency (e.g. means) or other basic estimates (e.g. regression coefficient) AND variation (e.g. standard deviation) or associated estimates of uncertainty (e.g. confidence intervals)
☐	☒	For null hypothesis testing, the test statistic (e.g. F, t, r) with confidence intervals, effect sizes, degrees of freedom and P value noted<br>*Give P values as exact values whenever suitable.*
☒	☐	For Bayesian analysis, information on the choice of priors and Markov chain Monte Carlo settings
☒	☐	For hierarchical and complex designs, identification of the appropriate level for tests and full reporting of outcomes
☐	☒	Estimates of effect sizes (e.g. Cohen's d, Pearson's r), indicating how they were calculated

Our web collection on statistics for biologists contains articles on many of the points above.

Software and code

Policy information about availability of computer code

Data collection	iEEG signals in mice were recorded using either the Cheetah 5.0 software (Neuralynx, USA) or the RHX software version 3.1 (Intan Technologies, USA). iEEG signals in human were recorded using XX. Video signal were captured using the OBS Studio software.<br><br>iEEG in human were recorded using the Pegasus software (Neuralynx, USA). To determine the exact location of each electrode contact, the MRI and postsurgical CT were co-registered using the Lead-DBS software (www.lead-dbs.org).
Data analysis	The human iEEG signals were preprocessed in Matlab (The MathWorks, Inc., US). All the remaining signal analysis and in silico stimulations were carried out using custom Python 3.6 scripts using the following libraries: dabest 2.13, matplotlib 3.3.4, mne 0.23.4, numpy 1.19.5, pandas 1.1.5, scikit-learn 0.24.2, scipy 1.5.4, seaborn 0.11.2.<br>Histology images were open in ImageJ 2.0.0. All plots were generated from python script and then compiled in Illustrator (Adobe Illustrator CC 2017). Some illustrations were made using Biorender (https://www.biorender.com/, Agreement number: ZJ25GOYNCW). Video editing was made using Adobe Premier Pro (Adobe Premiere Pro CC 2022).<br><br>Custom code used for this article is available at: https://doi.org/10.6084/m9.figshare.25305238

For manuscripts utilizing custom algorithms or software that are central to the research but not yet described in published literature, software must be made available to editors and reviewers. We strongly encourage code deposition in a community repository (e.g. GitHub). See the Nature Portfolio guidelines for submitting code & software for further information.

Data

Policy information about availability of data

All manuscripts must include a data availability statement. This statement should provide the following information, where applicable:

- Accession codes, unique identifiers, or web links for publicly available datasets
- A description of any restrictions on data availability
- For clinical datasets or third party data, please ensure that the statement adheres to our policy

The simulation, experimental and anonymised clinical data collected in this study will be made available upon acceptance as open data on the Bern Open Repository and Information System at https://boris-portal.unibe.ch/

The code will be openly published on github upon acceptance.

Research involving human participants, their data, or biological material

Policy information about studies with human participants or human data. See also policy information about sex, gender (identity/presentation), and sexual orientation and race, ethnicity and racism.

Reporting on sex and gender	We report self-reported gender of 10 participants (5 women, 5 men, 0 other) in the Extended Data Table 1. We did not perform any gender-based analysis.
Reporting on race, ethnicity, or other socially relevant groupings	We did not collect any race or ethnicity based informations
Population characteristics	See Extended Data Table 1.
Recruitment	Human data were collected from 10 patients (see Extended Data Table 1) with intractable epilepsy undergoing invasive presurgical evaluation with stereo-EEG at Inselspital, Bern, Switzerland.
Ethics oversight	This study was approved by the ethics committee of the Canton Bern (ID 2018-01387).

Note that full information on the approval of the study protocol must also be provided in the manuscript.

Field-specific reporting

Please select the one below that is the best fit for your research. If you are not sure, read the appropriate sections before making your selection.

☒ Life sciences ☐ Behavioural & social sciences ☐ Ecological, evolutionary & environmental sciences

For a reference copy of the document with all sections, see nature.com/documents/nr-reporting-summary-flat.pdf

Life sciences study design

All studies must disclose on these points even when the disclosure is negative.

Sample size	No sample size calculation was performed, but our sample sizes are similar to those reported in previous publications. We are confident that the sample size is sufficient since the main findings can be observed in longitudinal data of individual subjects.
Data exclusions	Mice with poor iEEG signal or no visible response to optogenetic stimulations were discarded from the beginning after a initial quality check evaluation and were not reported in the study (8 out of 42 mice tested, all 34 others mice are included in the manuscript).<br>Occasionally, injection of subconvulsive doses of PTZ (20mg/kg i.p. or less) still lead to seizure. In this case, data from the session were discarded and not included in the analysis (8 out of 31 subconvulsive PTZ sessions).
Replication	Mice experimentation was carrying out on six different batch, over a period of 3 years. Each experiment was replicated using two or more different batch. No difference were observed among different batch.
Randomization	Each experiment was designed as follow: each mice underwent one or more experimental block. A given block was composed of several sessions with different conditions (e.g. GABA-ergic modulation, stimulation frequency) performed in a randomized order, with an interval of 48-72h in-between sessions.<br>For human data, no randomization was possible as the Benzodiazepine was given for clinical reason. The same stimulation protocol was repeated before (baseline) and after (Benzodiazepine) the injection.
Blinding	Experimenter were not blind during data acquisition. All the signal preprocessing and analysis including semi-manual artefact rejections were done blind to the pharmacological condition. Video recordings of the induced seizures were scored offline and blinded to pharmacological condition.

Reporting for specific materials, systems and methods

We require information from authors about some types of materials, experimental systems and methods used in many studies. Here, indicate whether each material, system or method listed is relevant to your study. If you are not sure if a list item applies to your research, read the appropriate section before selecting a response.

Materials & experimental systems

n/a	Involved in the study	
☐	☒	Antibodies
☒	☐	Eukaryotic cell lines
☒	☐	Palaeontology and archaeology
☐	☒	Animals and other organisms
☐	☒	Clinical data
☒	☐	Dual use research of concern
☒	☐	Plants

Methods

n/a	Involved in the study	
☒	☐	ChIP-seq
☒	☐	Flow cytometry
☒	☐	MRI-based neuroimaging

Antibodies

Antibodies used	- Anti-GFP primary anti-body (1:5000, Ref. A10262, Invitrogen, USA)<br>- Goat Anti-Chicken IgY H&L (DyLight® 488) (1:500 Abacam, ab96947).
Validation	- For A10262, see https://www.citeab.com/antibodies/2401395-a10262-gfp-polyclonal-antibody/publications<br>- For ab96947, see https://www.citeab.com/antibodies/2360402-ab96947-goat-anti-chicken-igy-h-l-dylight-488<br>The combination of two have been previously used in several studies, see for example Facchin and al., J. Neuro. 2020.

Animals and other research organisms

Policy information about studies involving animals; ARRIVE guidelines recommended for reporting animal research, and Sex and Gender in Research

Laboratory animals	A total of 34 C57BL/6JRj and 4 PV_ires_Cre male mice aged between 2 and 4 months old were included in the study. Wild-type mice were ordered from Janvier Labs (https://janvier-labs.com/en/fiche_produit/2_c57bl-6jrj_mouse/). PV_ires_Cre mice come from the Jackson Laboratory (https://www.jax.org/strain/017320) and were bred a the central animal facility of the Department for BioMedical Research, University of Bern.
Wild animals	This study did not involve wild animals.
Reporting on sex	Ovarian cycle in mice is known to be a powerful modulator of seizure resilience and cortical excitability (see Maguire and al., Nat. Neuro. 2005). As our experimental design involved longitudinal recordings across several days, we only included male animals to avoid confounding effect.<br>Note that in the human data, as the experimental timeline is shorter we included both gender.
Field-collected samples	This study did not involve field-collected samples.
Ethics oversight	All experiments on mice were conducted in accordance with protocols approved by the veterinary office of the Canton of Bern, Switzerland (license no. BE 19/18 and BE 51/2022).

Note that full information on the approval of the study protocol must also be provided in the manuscript.

Clinical data

Policy information about clinical studies

All manuscripts should comply with the ICMJE guidelines for publication of clinical research and a completed CONSORT checklist must be included with all submissions.

Clinical trial registration	Not applicable, not a clinical trial. Reuse of human data.
Study protocol	This study was approved by the ethics committee of the Canton Bern (ID 2018-01387).
Data collection	Data was collected between September 2019 and December 2022 at the Inselspital, Bern University Hospital.
Outcomes	Not applicable, not a clinical trial. Reuse of human data.
